# Differences in muscle activity during hand-dexterity tasks between women with arthritis and a healthy reference group

**DOI:** 10.1186/1471-2474-15-154

**Published:** 2014-05-15

**Authors:** Sofia Brorsson, Anna Nilsdotter, Carina Thorstensson, Ann Bremander

**Affiliations:** 1 Health and Welfare, Dala Sports Academy, Dalarna University, SE-781 88 Falun, Sweden; 2 School of Business and Engineering, Department of Exercise Physiology, Biomechanics and Health, Halmstad University, Halmstad, Sweden; 3 Department of Research and Education, Halmstad County Hospital, Halmstad, Sweden; 4 Department of Clinical Neuroscience and Physiology, University of Gothenburg, Gothenburg, Sweden; 5 Department of Medicine, Solna, Karolinska Institutet, Stockholm, Sweden; 6 Department of Clinical Sciences, Lund, Section of Rheumatology, Lund University, Lund, Sweden; 7 Research and Development Center, Spenshult, Oskarstrom, Sweden

**Keywords:** Muscle activation, Muscle extension force, Flexion force, Female, Daily activities

## Abstract

**Background:**

Impaired hand function is common in patients with arthritis and it affects performance of daily activities; thus, hand exercises are recommended. There is little information on the extent to which the disease affects activation of the flexor and extensor muscles during these hand-dexterity tasks. The purpose of this study was to compare muscle activation during such tasks in subjects with arthritis and in a healthy reference group.

**Methods:**

Muscle activation was measured in m. extensor digitorium communis (EDC) and in m. flexor carpi radialis (FCR) with surface electromyography (EMG) in women with rheumatoid arthritis (RA, n = 20), hand osteoarthritis (HOA, n = 16) and in a healthy reference group (n = 20) during the performance of four daily activity tasks and four hand exercises. Maximal voluntary isometric contraction (MVIC) was measured to enable intermuscular comparisons, and muscle activation is presented as %MVIC.

**Results:**

The arthritis group used a higher %MVIC than the reference group in both FCR and EDC when cutting with a pair of scissors, pulling up a zipper and—for the EDC—also when writing with a pen and using a key (p < 0.02). The exercise “rolling dough with flat hands” required the lowest %MVIC and may be less effective in improving muscle strength.

**Conclusions:**

Women with arthritis tend to use higher levels of muscle activation in daily tasks than healthy women, and wrist extensors and flexors appear to be equally affected. It is important that hand training programs reflect real-life situations and focus also on extensor strength.

## Background

Impaired hand function is common in subjects with arthritis, and it affects daily activities and quality of life [1,2]. Reduced hand function occurs early during the course of the disease [3], and the impaired strength and dexterity affects many daily tasks [4]. Impaired hand grip function can be due to pain, to reduced muscle strength, and—at late stages in some patients—to hand deformity [5-8].

Exercise programs are commonly used to improve hand function, but there is little information on the types of exercise that are most effective. Hand exercises are often used to improve dexterity and hand flexion force. However, we have shown that people with rheumatoid arthritis (RA) have impaired finger extension force compared to healthy subjects [9], and there is also a lower correlation between grip force and finger extension force [10], indicating that exercises to improve the extension force may be important. As has been shown for lower extremity, trunk, and shoulder function, a balance between flexor and extensor muscle activation may be essential for optimal function in the hand and forearm [11,12].

In order to tailor exercise programs for improvement of hand dexterity and hand force, muscle activation must be studied. Strength measurements can quantify the effects of arthritis on the muscles, but they do not measure muscle fiber recruitment during daily hand tasks. The use of electromyography (EMG) can indicate whether there is a difference in recruitment of muscle fibers in subjects with arthritis and in healthy subjects. Only a few studies have been published on muscle activation in people with arthritis using EMG, and the results are inconclusive [13,14].

The aim of this study was to use EMG to measure muscle activation in flexor and extensor muscles of the wrist and fingers during hand-dexterity exercises in women with RA or hand osteoarthritis (HOA), and to compare the findings with those from a healthy reference group of women.

## Methods

### Subjects

The RA subjects recruited were diagnosed according to the 1987 ACR criteria [15]. They were enrolled consecutively from March through May 2011 when they visited a specialist outpatient clinic in the southwest of Sweden. The inclusion criteria for the RA patients were a disease duration of at least one year and full active finger extension and flexion ability. The exclusion criterion was primary hand surgery.

Female patients with HOA were identified from five primary healthcare units in the same geographic area in March through September 2011. The inclusion criteria were clinically diagnosed and symptomatic HOA and full active finger extension and flexion ability. The exclusion criteria were any rheumatic disease other than HOA and/or primary hand surgery.

The reference group was recruited using posters at Halmstad University and at Halmstad County Hospital during the period March through June 2011. In addition, the women with RA or HOA were also asked to bring friends who would be willing to participate in the study. The exclusion criteria for the reference group were inflammatory or muscle diseases, or previous hand or arm injuries.

The study was approved by the Ethics Committee of Lund University, Sweden. All procedures complied with the Declaration of Helsinki.

### Muscle activation

To assess muscle activation, the participants underwent surface electromyography (sEMG) following a standardized procedure. The basic approach is to collect sEMG data, which reflect the total muscle activation while subjects perform activities. The sEMG activity of m. extensor digitorum communis (EDC) and m. flexor carpi radialis (FCR) was measured in the dominant hand while performing four daily activities and four commonly used hand exercises.

The sEMG procedure started with cleaning of the skin using ethanol to minimize the impedance before two disposable (circular ø 10 mm), pre-filled Ag/AgCl Ambu blue sensor surface electrodes (Ambu A/S, Ballerup, Denmark) were attached over the muscle belly of the EDC and FCR and aligned with the direction of the fibers, and one reference electrode was attached over the ulnaris [16]. The ME6000 8-channel Biomonitor system (Mega Electronics Ltd., Kuopio, Finland) was used for sEMG measurements. Data were collected at a sampling frequency of 1,000 Hz.

To allow intermuscular comparisons, the maximal voluntary isometric contraction (MVIC) was measured and recorded with sEMG during a strength test performed on the two devices EX-it and Grippit. EX-it is a device for evaluation of finger extension force (in Newtons, N) [9] while Grippit measures grip force (Detektor AB, Göteborg, Sweden) [17]. Both devices have shown good validity and reliability [9,17]. The procedure for the measurements was standardized in terms of sitting position, verbal instructions, and encouragement [18,19] and it was carried out by one assessor only.

### Hand exercises and daily tasks

**Figure 1:**
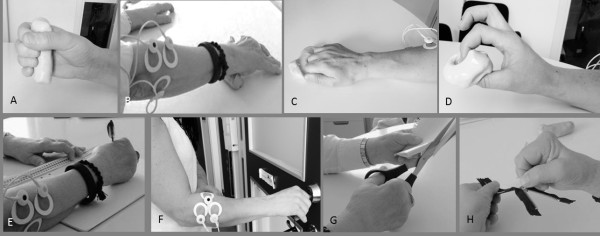
**Muscle activation was measured with surface EMG in m. extensor digitorum communis and m. flexor carpi radialis in the dominant hand while performing four hand exercises and four daily tasks: (a) squeezing the putty, (b) rolling the putty with a flat hand, (c) finger extension, (d) isolated opposition, digits II–V (daily activities), (e) writing with a pen, (f) locking a door with a key, (g) cutting with scissors, and (h) pulling up a zip. ***Consent was obtained from individuals for publication of the images*.

Muscle activation was measured during four exercises from a programme using silicone rubber (“therapeutic putty”) to improve hand function: squeezing the putty, rolling the putty with a flat hand, finger extension, and isolated opposition, digits II–V (Figure 1a-d). Muscle activation was also measured with sEMG during four commonly performed tasks: writing with a pen, locking a door with a key, cutting with scissors, and pulling up a zipper (Figure 1e-h). All the tasks were first demonstrated to the participants and then they were allowed to familiarize themselves with the task; after that, the performances were recorded. All tasks were standardized regarding instructions and completion.

### Self-reported hand function

The subjects scored their hand function with the outcome measure Quick Disabilities of Arm, Shoulder, and Hand (Quick DASH). The Quick DASH measure has two components: the disability/symptom section (11 items) and the optional high-performance sport/music or work section (4 items) (http://www.dash.iwh.on.ca). In this study, the Swedish version of the disability/symptom section was used [20]. The items are rated from 1 (no difficulty) to 5 (unable to perform). A score from 0 to 100 is calculated, and a higher score indicates greater disability [7]. The subjects marked their pain and stiffness during the previous week on a visual analogue scale (VAS; 0–100 mm) with the endpoints no pain/stiffness (0 mm) and pain/stiffness as bad as it can be (100 mm). All self-reported measures were collected on the same day as the muscle tests were performed.

### Data and statistical analysis

Values from the maximal flexion force and maximal extension force were normalized and expressed as a percentage of the MVC, allowing intermuscular comparisons to be made [13]. The raw sEMG signal (transformed with root mean squared average) from the different hand-dexterity tasks was divided by the sEMG signal from the EX-it and Grippit trials for maximal isometric contraction, expressed as %MVIC, and processed with Megawin software (Mega Electronics). A high percentage of MVIC indicates high muscle activation in extension muscles (EDC) and/or flexion muscles (FCR). Values were obtained from the first MVC trial of each subject.

Descriptive data are presented as mean and 95% confidence interval (95% CI) or as median and 25–75 percentiles based on information on how normally distributed the data were. Differences between three groups were analyzed with the Kruskal-Wallis test but due to the limited number of subjects, we also analyzed differences between the arthritis group (RA and HOA) and the reference group using the Mann–Whitney test. A p-value of 0.05 or less was considered to be statistically significant. SPSS version 20.0 for Windows was used for statistical analysis.

## Results

### Subjects

Data were analyzed from 20 subjects with RA, from 16 with HOA, and from 20 subjects in the reference group (RG). One subject with HOA had to be excluded due to a change of diagnosis during the study (from HOA to unspecified arthritis), and data from three subjects with HOA were lost due to technical problems.

Some differences were found between the three groups. On average, the reference group was slightly younger than the other two groups, and the RA group was younger than the HOA group. The differences found between the RA group and the HOA group were in the pain score, where the RA group reported less pain than the HOA group (p = 0.01), but a larger proportion of the subjects with RA were more often on medication (NSAIDs or disease modifying drugs (DMARDs)). Both the RA subjects and the HOA subjects had a significantly lower maximal extension and flexion force than the reference group (p < 0.001) (Table 1).

**Table 1:** Subject characteristics, disability, and force measurements in the rheumatoid arthritis (RA), hand osteoarthritis (HOA), and healthy groups

***Sociodemographic***	***RA *(*n *= *20*)**	***HOA *(*n *= *16*)**	***Healthy *(*n *= *20*)**
Age (years) m^1^ (95% CI)	59.5 (54–64)	68.1 (62–72)	56.0 (51–60)
Disease duration (years) m (95% CI)	20.0 (15.1–28.2)	15.0 (11.5–20.3)	Na
Medication* (%)	100	41.1	Na
***Measurements of disability***			
Quick DASH^2^ m (95% CI)	40.9 (33.2–50.7)	31.8 (30.8–45.2)	2.3 (0.9–10.5)
VAS stiffness^3^ m (95% CI)	3.0 (2.0–4.1)	4.0 (2.5–5.1)	Na
VAS pain^3^ m (95% CI)	2.0 (1.5–3.0)	3.9 (3.1–5.0)	Na
***Hand force *(*N*)**			
Max extension (N)^4^ m (95% CI)	20.0 (17.8–26.1)	26.0 (21.4–31.5)	33.5 (30.6–39.3)
Max flexion (N)^5^ m (95% CI)	81.0 (67.4–137.4)	81.5 (70.8–125.8)	245.0 (195.3–275.6)

^1^Mean with 95% Confidence interval.

^2^QuickDASH: Disability in arm shoulder and hand, 0–100 best to worst.

^3^VAS stiffness and VAS pain, 0–10cm, best to worst.

^4^Measured with EX-it, value in Newtons (N).

^5^Measured with Grippit, value in Newtons (N).

*Self-reported medication, painkillers or disease modifying drugs (RA only).

### Muscle activation in daily activities and differences between groups

**Figure 2:**
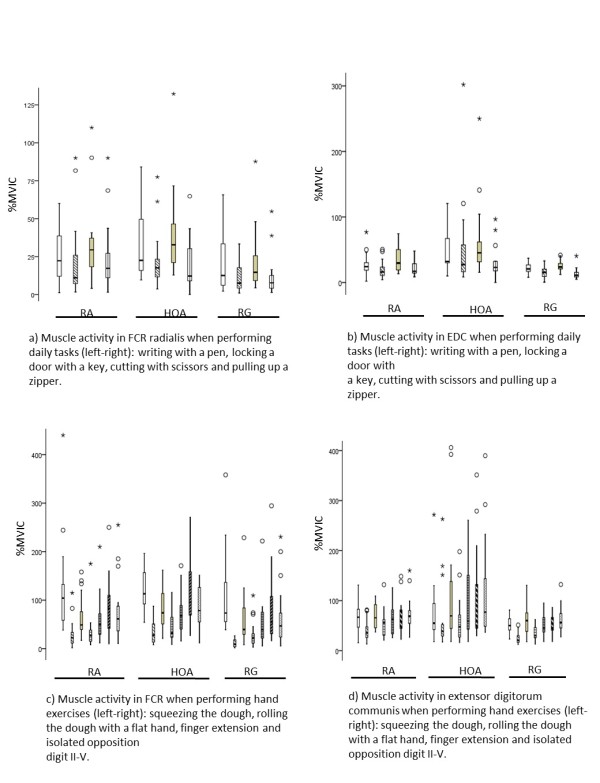
Muscle activation in m. flexor carpi radialis (FCR) and m. extensor digitorum communis (EDC) when performing daily activities and hand exercises for patients with reumatoid arthritis (RA), hand osteoarthritis (HOA) and a healthy reference group (RG) presented as percentage of maximal voluntary isometric contraction (%MVIC) (box plot shows median and range; * and º show outliers). a) Muscle activity in FCR radialis when performing daily tasks (left-right): writing with a pen, locking a door with a key, cutting with scissors and pulling up a zipper. **b)** Muscle activity in EDC when performing daily tasks (left-right): writing with a pen, locking a door with a key, cutting with scissors and pulling up a zipper. **c)** Muscle activity in FCR when performing hand exercises (left-right): squeezing the dough, rolling the dough with a flat hand, finger extension and isolated opposition digit II-V. **d)** Muscle activity in extensor digitorum communis when performing hand exercises (left-right): squeezing the dough, rolling the dough with a flat hand, finger extension and isolated opposition digit II-V.

The daily activity tasks found to have the highest muscle activation in all groups (expressed as %MVIC), i.e. involving the EDC and the FCR to the greatest extent, were cutting with scissors and writing with a pen (Table 2 and Figure 2). There was a difference between the arthritis group and the reference group—suggesting a higher %MVIC for both flexors and extensors in the arthritis group—while cutting with scissors, pulling up a zip, and (for the extensors) when writing with a pen (p < 0.02). These differences were maintained for comparisons between the HOA group and the reference group (p ≤ 0.05), while significant differences between the RA group and the reference group were only found for pulling up a zip and cutting with scissors (flexor activity, p = 0.04) (Table 2).

**Table 2:** Muscle activity in extensor digitorum communis (EDC) and flexor carpi radialis (FCR) during daily activities and hand exercises for patients with reumatoid arthritis (RA), hand osteoarthritis (HOA) and a healthy reference group (RG) presented as % of MVIC (median and 25–75 percentiles)

	**RA (n = 20)**		**HOA (n = 16)**		**RG (n = 20)**	
	**% *of MVC in EDC***	**% *of MVC in FCR***	**% *of MVC in EDC***	**% *of MVC in FCR***	**% *of MVC in EDC***	**% *of MVC in FCR***
***Daily activities***						
**Task 1**(pen)	24.2 (18.0–30.5)	22.3 (11.4–40.6)	32.3** (30.1–75.0)	22.5 (15.8–53.8)	20.8* (16.9–27.4)	12.6 (5.5–35.8)
**Task 2** (locking)	16.3 (10.7–23.7)	11.0 (6.4–26.0)	27.4* (15.3–65.7)	17.6 (11.3–25.5)	15.5 (8.3–21.0)	7.5 (4.1–18.0)
**Task 3** (cutting)	29.6 (19.0–51.1)	29.5* (18.0–37.5)	45.4*** (30.6–62.0)	32.8** (20.9–49.3)	23.7** (20.7–29.6)	14.7*** (9.2–25.7)
**Task 4** (zipper)	16.9* (14.2–30.3)	17.3** (10.9–27.3)	23.0** (17.2–32.4)	12.2 (8.9–30.3)	11.0*** (8.5–15.7)	7.7** (4.0–12.7)
***Hand exercises***						
1 (squeezing)	66.8 (45.8–85.3)	104.2 (58.2–132.6)	54.9 (41.0–94.9)	113.1 (89.4–159.1)	50.1 (41.1–63.7)	73.5 (55.0–137.0)
2 (rolling)	35.5* (26.1–48.4)	22.6* (11.3–36.4)	38.7** (28.1–53.5)	28.0** (13.3–53.7)	20.9*** (16.5-30.5)	9.3*** (4.1-18.7)
3 (finger ext)	65.6 (44.1–93.0)	49.1 (39.6–76.2)	69.6 (44.0–138.8)	73.9 (49.1–116.6)	60.1 (36.7–77.9)	39.4 (29.2–84.6)
4a (opposition II)	55.8 (29.9–62.2)	27.2 (15.1–38.6)	47.6 (27.1–74.3)	32.1 (22.5–65.6)	29.9* (25.9–46.6)	21.9 (10.3–33.1)
4b (opposition III)	62.5 (33.1-85.7)	50.1 (29.0-73.4)	59.3 (39.9-169-5)	68.0 (38.9-89.4)	45.2 (36.4–66.3)	39.1 (20.9–77.9)
4c (opposition IV)	58.2 (44.2–82.9)	82.8 (39.9–110.7)	57.7 (42.8–134.2)	95.6 (69.0–160.9)	45.8* (40.8–67.2)	55.1 (30.7–108.9)
4d (opposition V)	68.9 (54.0–80.4)	61.3 (36.7–89.0)	77.1 (47.9–163.8)	78.4 (54.7–129.3)	56.1 (45.3–77.5)	46.9 (22.1–76.8)

Comparisons between RA, HOA and the reference group (RG) in %MVIC of muscle activation analyzed by Kruskal-Wallis for differences between three groups (results after RA or HOA). Differences between arthritis group (RA and HOA) and RG was analyzed by Mann–Whitney (results after the RG).

Daily activities: task 1 = writing with a pen, task 2 = locking a door with a key, task 3 = cutting with scissors, task 4 = pulling up a zipper.

Hand exercises: 1 = squeezing the dough, 2 = rolling the dough with flat hands, 3 = finger extension, 4 = isolated opposition, digits II–V.

*p < 0.05, **p < 0.01, ***p < 0.001.

### Muscle activation in hand exercises

In the hand exercises, the highest %MVIC value for the EDC muscle was found in the exercise finger extension and isolated opposition. For the FCR muscle, the exercises of squeezing the putty and isolated opposition gave the highest %MVIC. The commonly performed hand exercise "rolling dough with flat hands" required the lowest %MVIC in all subjects, and may be less effective if the aim is to improve muscle strength (Table 2 and Figure 2).

## Discussion

Subjects with arthritis were weaker in terms of both extension and flexion force compared to healthy subjects, and they also tended to use a higher amount of muscle activation in all the daily activity tasks tested—in both extensor muscles and flexor muscles of the hand. Little is known about how much muscle activation of the maximal isometric muscle force subjects with arthritis use to perform daily activities compared to healthy subjects. The present study showed that cutting with scissors and writing with a pen required the highest muscle activation for finger flexor and extensor muscles in all subjects, and we found that the muscle activation was consistently higher in subjects with arthritis than in the reference group.

Hand strength and hand function can be improved with exercise [21-23], but hand strengthening exercises most often focus on grip force and little attention is paid to extension force. We suggest that hand exercise programs should be designed to improve the strength of both flexor and extensor muscles of the forearm and that both outcomes should be measured to monitor changes over time.

There is no doubt about the importance of the extensor muscles of the forearm in hand tasks, and in an earlier study we found that there was a higher correlation between hand flexion and extension strength in healthy women than in women suffering from arthritis [10]. Post hoc analysis revealed that this was also true of subjects with HOA compared to the reference group in that study. This emphasizes the importance of further research on whether strengthening exercises not only improve hand strength and hand function but also affect the relationship between the agonist and antagonist muscles of the forearm in subjects with arthritis to a similar extent to those in healthy subjects.

This study has also contributed with new information concerning muscle activity in forearm extensors and flexors, which are used by subjects with arthritis to perform a number of daily activities. Concentrating on extension force and extension muscle activation is fairly new in the field of arthritis research, and there have been few hand muscle activation measurements derived from sEMG in subjects with arthritis. Calder et al. [13] studied muscle activation in forearm muscles during daily activities in a small cohort of women with and without HOA, and found a tendency of impaired muscle function in the women with HOA [13]. In a study by de Olivera et al. [24], subjects with HOA were found to have impaired grip force control when lifting an object compared to healthy individuals [24]. This is in agreement with the findings from the present study, in which women with HOA were just as affected by the disease as the women with RA were, and required higher muscle activation in hand extensors and flexors compared to the reference group. Improvement of hand strength is just as important in subjects with HOA as in subjects with RA [25-27].

Patients with both RA and HOA are most often affected early during the course of disease. Hand strength should therefore be assessed and treated at an early stage [28,29]. In order to design an exercise programme to improve general hand strength, we need more information on what muscle activation the different exercises generate, and the present study contributes with such information. If one is aiming for improved strength, exercises requiring a higher %MVIC might be better to include in a programme than rolling the putty (exercise task 2) with flat hands, which required a much lower %MVIC.

This study had several limitations. Firstly, we used a device that measures finger extension force while flexion force was measured as grip force, which must be taken into consideration when comparing the two measurements. In future studies, we also recommend measurement of wrist extension force, to further explore the amount of force required and the amount of muscle activation in subjects with impaired hand strength. Secondly, the small sample size in this study was a limitation, which may have led to type-II error. However, since there have been few earlier studies using sEMG on subjects with RA or HOA, the results can be used when designing larger studies. Both RA and HOA are more common in women than in men, with a gender ratio of 3:1 and a 2:1, respectively [30]. There is also a well-known difference between muscle strength in men and in women [31], which is why this study was performed on women.

There was a trend for the reference tests to not represent the maximum value compared to values registered in the exercises and the daily activities for patients with RA and HOA. One reason for this might be pain, another could be that the soft dough allowed an increased range of motion which resulted in increased strength. Earlier studies have found that isometric normalization contractions can result in normalized sEMG values greater than 100%. In a study by Clarys et al. normalized EMG over 160% MVIC was reported for the triceps brachii during swimming [32].

In general, people with arthritis have an impaired hand strength compared with healthy subjects [17,21,31,33]. As a mean, the arthritis patients performed 69% of the healthy subjects’ finger extension strength and 33% of their grip strength. Earlier studies have found that subjects with HOA and RA only have 20% - 50% of healthy subjects’ grip strength, depending on disease duration, pain and hand deformity [13,17]. The grip strength found in the healthy subjects in this study is in agreement with normal values earlier published [34,35]. We found that healthy people used greater force during the tests than arthritis patients.

Furthermore, the force used was at a considerably lower percentage relative to their maximum force than when the tests were performed by patients with arthritis.

Healthy people might use greater force when writing with a pen because it is possible (but not necessary), while people with arthritis might only use the necessary force required by the task. Other studies exploring sEMG in subjects with arthritis during dexterity tasks are lacking making comparisons hard. A study by Calder et al. showed no difference between healthy subjects and subjects with hand OA performing a dexterity test [13].

Our inclusion criteria for RA and HOA did not include impaired hand function. The reason was that we were interested in the diagnosis and its effect on hand function, not necessarily studying subjects known to have impaired hand function. Earlier reports have stated that hand function is affected in most patients with RA [36] and that it develops early in the course of the disease [37]. The self-reported DASH score ranged from 13.6 to 70.5 in the RA/HOA patients and from 0 to 5.7 in healthy subjects, which clearly indicates that hand function was affected in the subjects with arthritis in this study. However, hand deformity or nerve damage, which can be present in RA, was not an issue in this study. All the patients were able to perform the two strength tests according to the standardized manual and none of the subjects stopped the strength tests due to pain. However, it is impossible to know whether pain affected the muscle force and the muscle activation since many subjects with arthritis constantly experience pain in their hands. Our aim was to compare muscle activation in subjects with a disease affecting the hands and in healthy subjects, as reflected in “real life”.

## Conclusion

Women with arthritis tend to use higher levels of muscle activation in daily tasks than healthy women, and wrist extensors and flexors appear to be equally affected. We recommend that hand training programs should reflect real-life situations and also focus on extensor strength. However, it is important to take into consideration other aspects of pathology, such as range of motion and pain. Consequently, both hand flexion force and hand extension force should be measured and monitored over time to gain a full understanding of impaired hand function in subjects with arthritis.

## Competing interests

The authors declare that they have no competing interests.

## Authors’ contributions

SB, AN, CT, and AB made substantial contributions to the conception and design of the study. SB and AB performed the analysis and interpretation of data. SB, AB, CT, and AN were involved in drafting the manuscript and in revising it critically for important intellectual content. All authors read and approved the final manuscript.

## Pre-publication history

The pre-publication history for this paper can be accessed here:

http://www.biomedcentral.com/1471-2474/15/154/prepub
